# Oral health and functional capacity of centenarians

**DOI:** 10.1038/s41598-020-78842-w

**Published:** 2020-12-17

**Authors:** Caroline Sekundo, Eva Langowski, Samuel Kilian, Cornelia Frese

**Affiliations:** 1 Clinic for Oral, Dental and Maxillofacial Diseases, Department of Conservative Dentistry, University Hospital Heidelberg, Im Neuenheimer Feld 400, 69120 Heidelberg, Germany; 2 Institute of Medical Biometry and Informatics, University of Heidelberg, Heidelberg, Germany

**Keywords:** Medical research, Epidemiology

## Abstract

The number of very old individuals, namely centenarians, is growing fast. In dentistry, the increasing number of older adults retaining natural teeth present new challenges for preventive and restorative dental care. However, there is a considerable lack of knowledge on the oral health status and needs in this exceptional age group. The aim of this population-based study was to identify the prevalence of oral diseases, therapeutic needs and functional capacity (evaluating centenarians’ autonomy and their capabilities regarding treatment and oral hygiene) in centenarians. Subjects born before 1920 were recruited from population registries in South-Western Germany, providing information on dental health experiences, oral health behaviors and undergoing dental examination. 55 centenarians participated in the study (mean age ± SD = 101.2 ± 1.6, 83.6% females). Results were compared to epidemiological data on adults aged 75–100 years examined in the Fifth German Oral Health Study. Adherence to recommended dental behaviors and dental check-ups was lower in the centenarian population. Moreover, with the exception of a lower Root Caries Index, centenarians showed a higher caries experience, and presented with a mean DMFT of 25.2 ± 3.9, a DMFS of 111.0 ± 21.8, a root caries prevalence of 34.5% and a Restorative Index of 54.0%. Centenarians’ functional capacity was also considerably lower. Non-existent or greatly reduced treatment capabilities and oral hygiene capabilities were registered in 63.7% and 43.6% of cases, respectively. Centenarians with a lower educational level (*p* = 0.018), in a care facility (*p* = 0.045) or in need of nursing care (*p* = 0.001) were more likely to have a low functional capacity. 98.2% of centenarians received help in their daily activities but only 12.7% in their oral hygiene. In conclusion, although most still have natural teeth, a decline of oral health can be perceived. As compliance with recommended behaviors is limited and most centenarians can no longer undergo dental treatment, the lack of assistance in daily oral health care is problematic.

## Introduction

Demographic changes in high-income countries have led to an increasing population of older adults. Centenarians and supercentenarians are the oldest old within this group, and their number is growing rapidly. In 2018, about 14,000 persons born 1918 or earlier were registered in Germany. By 2028, predictions by the Federal Statistical Office of Germany estimate that numbers will have doubled, reaching 28,000, and will have doubled again by 2038, reaching 56,000^1^.

Nationally representative data from the German National Oral Health Surveys has shown a considerable decline in caries experience in older adults. The number of teeth lost due to caries has reduced significantly by 6.5 teeth in those aged 65–74 years between 1997 and 2014^2–4^. Even in 75–100-year-olds, examined for the first time in 2014, the mean number of natural teeth was 10.2^4^. Therefore, it is likely to assume that centenarians possess more teeth than ever before. Given the fact that their number is rising, valid epidemiological data on oral health and functional capacity in this high age group are urgently required. In light of the fact that dental diseases are mostly preventable and treatable, this information could also aid further measures to improve oral health in older adults.

However, studies on those aged 100 years or older have yet been confined to the field of medicine. Here, a considerable amount of research has been put forward to analyzing the secrets of centenarians’ longevity and health, given that they have outlived most of their birth cohort. For one thing, this is attributed to steadily improving life expectancies of all adults, as better health care and hygiene have led to longer lives^5,6^. However, this process has also increased aging and age‐related morbidity remarkably, and it is argued at the same time that the quality of life has not improved accordingly^7^. Some have reported high morbidity and poor functional health, stating that centenarians experience a multitude of chronic (not life-threatening) diseases and pain experience is frequent^8,9^. Conversely, many centenarian studies have stated that participants exhibited medical histories with remarkably low incidence rates of age-related diseases. Moreover, a delay in the onset of disease and a distinct compression of morbidity towards their life’s end has been reported. Many remain independent in daily living until their mid-90s^10–12^, and most report a high quality of life^13,14^.

Only few studies have yet examined centenarians’ oral health^15^. Based on self-reported information from the New England Centenarian Study (NECS) population, recruited throughout the US, a study by Kaufman et al. focuses on the number of teeth and edentulous rate^16^. In a second Chinese study, performed in the rural areas of the Xinjiang Uygur autonomous region, mucosal conditions of centenarians were reported, however, no further data regarding the oral status were provided^17^. The studies could show that the examined centenarians presented with good oral mucosal conditions^17^ and a relatively low edentulous rate in contrast to their birth cohort when aged 65–84 years old (36.5% vs. 46%)^16^. In contrast, a recent study by Beker et al., analyzing self-reports by centenarians recruited throughout the Netherlands via different media, reported an extremely high self-reported edentulous rate of 83%^18^. To date, worldwide, no clinical study has yet been carried out to assess centenarians’ oral health status in a representative study sample.

To the best of our knowledge, this is the first regional population-based study on the epidemiology of centenarians’ oral health in Germany. This is the first step in order to (1) identify the prevalence of dental diseases, therapeutic needs, as well as possible detrimental or beneficial factors in maintaining good oral health at high age and (2) explore age-related trends by comparison with national epidemiologic data on adults aged 75–100 years old. For an over-aging society, this information is crucial to assess needs in caries prevention and treatment.

## Methods

A cross-sectional survey and clinical examination were performed among centenarians living in South-Western Germany. Due to the exploratory nature of the study and in view of sample sizes reached by previously published studies on centenarians^8,16,19^, the target for patient recruitment was set at 50 study participants. The study (Heidelberg Dental Centenarian Study, HD-100Z) was approved by the Ethics Committee of the Medical Faculty of Heidelberg (S-168/2019) and registered with the German Clinical Trials Register (DRKS 00017128, date of registration: 20/05/2019) that is linked to the WHO clinical trials register. Informed written consent was obtained from all study participants. This study followed the Strengthening the Reporting of Observational Studies in Epidemiology (STROBE) guidelines^20^.

The catchment area extended from Karlsruhe in the south to Darmstadt in the north and from the Rhine-Palatinate to the Neckar-Odenwald district in its east–west extension. In order to recruit a study sample as unbiased and representative of the regional population as possible, all 183 registries were asked for contact information on every registered person born 1919 or earlier in April/May 2019. All 477 registered individuals were invited for study participation; three contact attempts were made. No initial exclusion criteria were applied. Fifty-five persons had already deceased or were no longer living under the registered address at the time of contact, resulting in a quality-neutral non-response of 11.5%. Of the 422 valid contacts, 117 refused participation and no contact could be established in 250 cases. The main reasons for refusing study participation was concern the visit would be too exhausting (55.6%), no interest (17.9%), cognitive restrictions/dementia (12.8%), poor physical health (12.0%) and other reasons (1.7%).

Accordingly, 55 centenarians participated and were interviewed and examined at their residence (private home or elder care facility) between May and October 2019.

Before entering the interview or examination, a shortened version of the Mini-Mental State Examination (short MMSE, max. 21 points)^21^ was performed. It can be assumed from previous centenarian studies^22,23^ that these MMSE items are unlikely to be biased by the impaired sensory functioning highly prevalent in this age group^24^. A score below 5 was considered as termination criterion. At a score between 5 and 10, the clinical examination was performed but no interview took place. However, information on dental visits, medical records, officially recognized care levels or degrees of disability etc. were registered if available by primary contacts or nursing staff.

The following outcome measures were assessed by interview and proxy information: (1) sociodemographic characteristics, (2) dental health experiences and (3) oral-health related behaviors. Regarding sociodemographic items, we used the standardized sociodemographic survey for older adults aged 75–100 years used in the 5th German Oral Health Study (DMS V)^4^, which also includes questions on the frequency of oral hygiene measures and visits to the dentists, the availability of dental care in care facilities, functional limitations to daily activities as well as disability and care levels. The assessment of educational levels was based upon participants’ highest obtained school degree, analogous to the DMS V (low = no degree obtained or basic track, medium = intermediate track, high = academic track). Care and disability levels are defined by German legislation^25–27^. The Degree of disability (GdB) is decided upon by the social security office; it measures the degree of impairment caused by a disability and ranges between 20 (low disability) and 100 (high disability)^26,27^. Care levels are assessed by the medical service of the statutory health insurance funds. They are classified into five nursing care levels^25^, with 1 representing a low level of impairment and five representing the highest level of impairment. Assessments include six areas of day-to-day life: mobility, mental and communicative skills, behaviors and psychological problems (i.e. fear, aggression), self-sufficiency, independent handling of illness or therapy-related demands (i.e. medication intake) and everyday life/ social contacts.

Subsequently, a dental and oral examination was conducted with binocular loupes and additional light source. Caries experience was recorded by means of the Decayed, Missing and Filled Teeth Index (DMF-T) and on the surface level by means of the Decayed, Missing and Filled Surfaces Index (DMF-S), according to WHO basic methods^28^. The D component includes carious teeth (or surfaces) with and without permanent restorations. The M component comprises all teeth missing for any reason. The F component includes teeth (or surfaces) with permanent restorations and without caries. Restorations serving as fixed dental prosthesis abutments as well as special crowns or veneers placed for reasons other than caries are not included.

The Restorative Index (RI)^29^ was calculated by the ratio of filled teeth to decayed teeth plus filled teeth (F/[D + F] × 100). The FST Index describes the number of functioning teeth by adding filled (F) and sound (S) teeth^30,31^. The Root Caries Index (RCI) was used to report root caries data. It describes the ratio of the number of teeth with carious lesions of the root and restorations of the root to the number of teeth with exposed root surfaces^32^. For further information regarding periodontal and peri-implant examinations on dentate participants, please see our previous publication^33^.

Last, in order to measure centenarians’ capacity and resilience to cope with their oral health needs, we evaluated their functional capacity as first described by Nitschke et al.^34^. This measure has successfully been implemented in the DMS V study. To this end, defined criteria regarding the capability of treatment, capability of oral hygiene and the centenarians’ autonomy were evaluated, using predefined fictitious scenarios. The evaluation was performed by the dental examiner after having received all self-reported and proxy-reported information and completed all other cognitive and clinical assessments. Capability of treatment was defined as the centenarian’s capability to partake in dental treatment procedures. The capability of oral hygiene described his or her ability to participate in a dental prophylaxis session, understand and implement oral hygiene instructions. Autonomy described the centenarian’s capability to decide to call on a dentist and organize the visit (i.e. by calling a taxi or asking a relative or care personnel to drive him/her to the dental practice; it was not necessary for the centenarian to journey unaided). These aspects where then translated into four resilience levels (1 = high functional capacity, 4 = low functional capacity).

Data analysis was performed with R 3.6.3.^35^ and SPSS, Version 24.0^36^. Characteristics of the study population were analyzed by means of descriptive statistics and compared to existing population data from the 5th German Oral Health Study (DMS V)^4^ on adults aged 75–100 years (n = 1133, minor variations possible due to non-response in some categories). Differences between the two groups are purely descriptive, statistical testing could not be performed due to the unavailability of DMS V raw data. Mean ± SD of continuous variables and proportion and frequency of categories of factor variables are reported. Associations between dental variables and gender, educational level, type of residence, utilization of dental services and the presence of a nursing care level were analyzed using the Kruskal–Wallis test. *p* values are purely descriptive and regarded significant if ≤ 0.05.

### Ethical approval

The study was approved by the local Medical Ethics Committee (S-168/2019) and performed in accordance with the World Medical Association Declaration of Helsinki. Informed written consent was obtained from all study participants.

## Results

Table 1 presents age, sex, social status, as well as recognized disability, nursing care levels and place of residence of the study population as well as those aged 75–84 and 85–100 years in Germany (DMS V^4^). Examined centenarians had a mean age of 101.2 ± 1.6 years, were mostly female (83.6%) and had attained a poor or medium education level. Although a majority (87.3%) was in need of care, more than half were still living at home. A medium care level (3/5) was most frequent. A MMSE score ≤ 10 was registered in 12 centenarians (21.8%), who were thus not questioned about their personal responsibility for their dental health. The compared group of 75–100-year-olds from the DMS V study had a mean age of 81.2 ± 5.1 years, 75% were between 75 and 84 years old and 25% were between 85 and 100 years old. Therefore, the age of the centenarian group (100+) of this study lies approximately 20 years above the mean age of the DMS group. However, due to the regional limitation and the non-response bias of the centenarian group, all comparisons between the two age groups must be interpreted with caution.

**Table 1:**
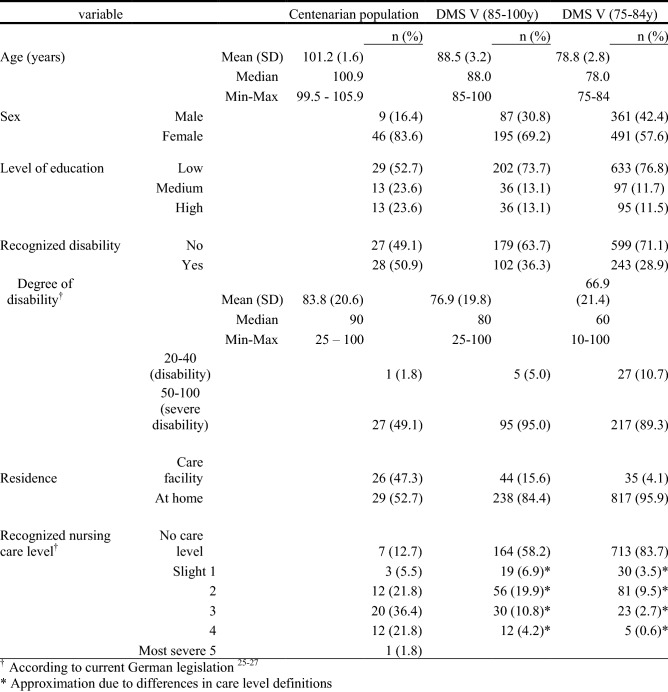
Characteristics of study populations.

### Dental health behaviors and beliefs

Adherence to recommended dental behaviors was lower in the centenarian population compared to the 75–100-year-olds examined in the DMS V. Only 34.9% thought that much could be done oneself to preserve dental health, whereas 34.9% showed a medium personal responsibility for their dental health and 30.2% showed low responsibility. 50.9% of centenarians brushed their teeth or dentures the recommended amount of 2–3 times daily, compared to 75.5% in the DMS V 75–100-year-olds. Similarly, only 50.9% had visited (or been visited) by the dentist in the past year (vs. 73.9% of the 75–100-year-olds), 29.1% had not seen a dentist for over 5 years. The ratio of participants with a complaint-oriented approach to seeking out a dentist (vs. a control-oriented approach) increased compared to the DMS V group (see online Appendix Supplementary Table S1 for details on the different age groups).

### Caries experience

The mean DMF-T of centenarians was 25.2 ± 3.9, the greatest proportion being the mean of 22 ± 7.2 missing teeth M(T). Twenty centenarians were edentulous (36.4%, M(T) = 28), all had been fitted with full dentures. In comparison, 32.8% of the 75–100-year-olds examined in the DMS V were edentulous. However, two centenarians had lost only few teeth and kept their permanent dentition without any kind of artificial teeth (M(T) = 5 and 6, respectively). The DMF-T of men was lower than in women (21.8 vs. 25.8, *p* = 0.006). Supplementary Fig. S1 (see online Appendix) shows an exemplary clinical case of a 102-year old male participant. However, DMF-T differences in educational level, residence (at home vs. care facility), utilization of dental services (control-oriented vs. complaint-oriented) or in the presence of a recognized nursing care level were not statistically significant.

**Figure 1:**
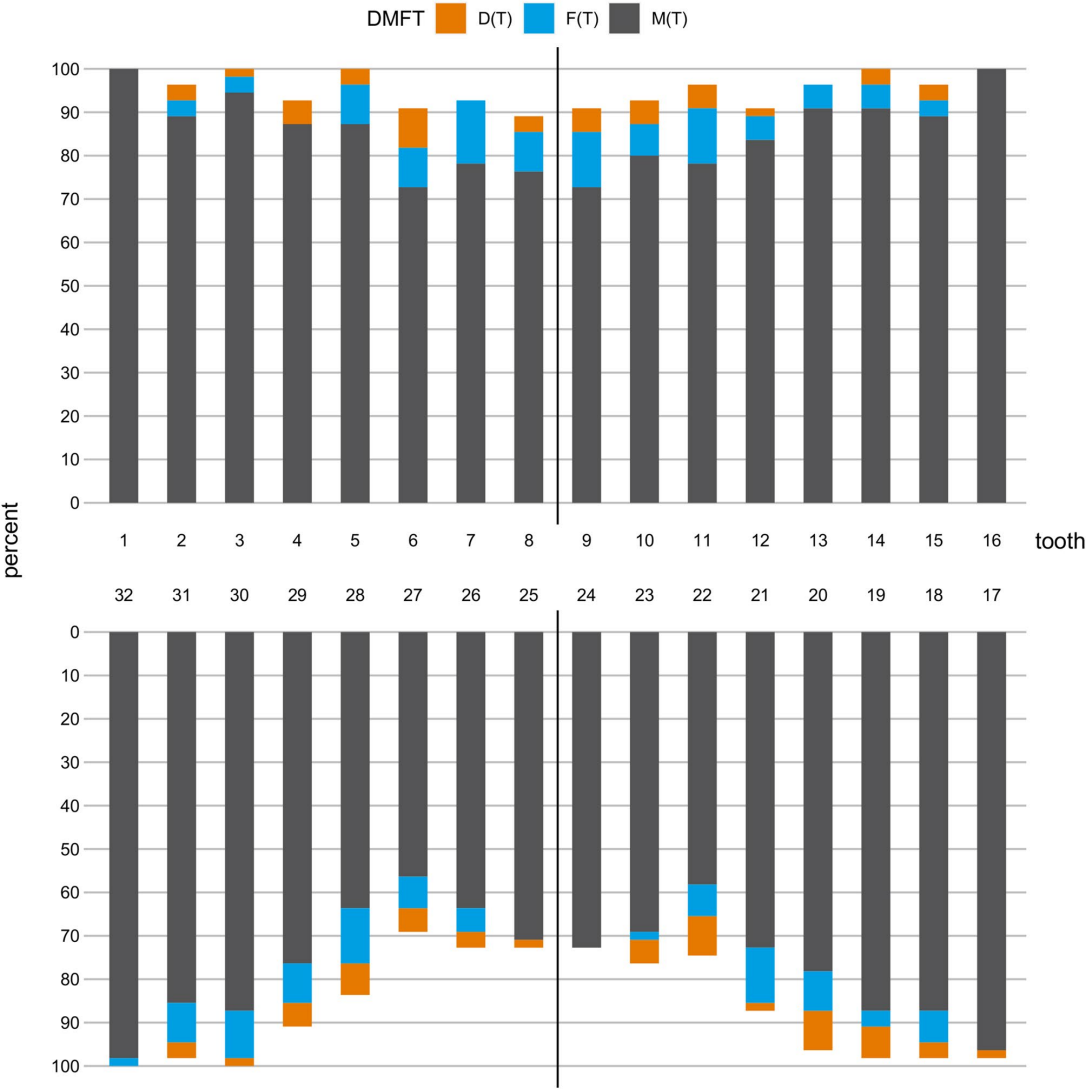
Distribution of Decayed (D), Missing (M) and Filled (F) teeth in centenarians (n = 55).

The distribution of decayed, missing and filled teeth in the centenarian population is shown in Fig. 1. Posterior teeth were more likely to be missing than anterior teeth, and maxillary teeth were more likely to be missing than mandibular teeth. Lowest DMF-T values were found in the anterior mandibular teeth. There were no considerable differences in the distribution of caries or intact restorations.

**Figure 2:**
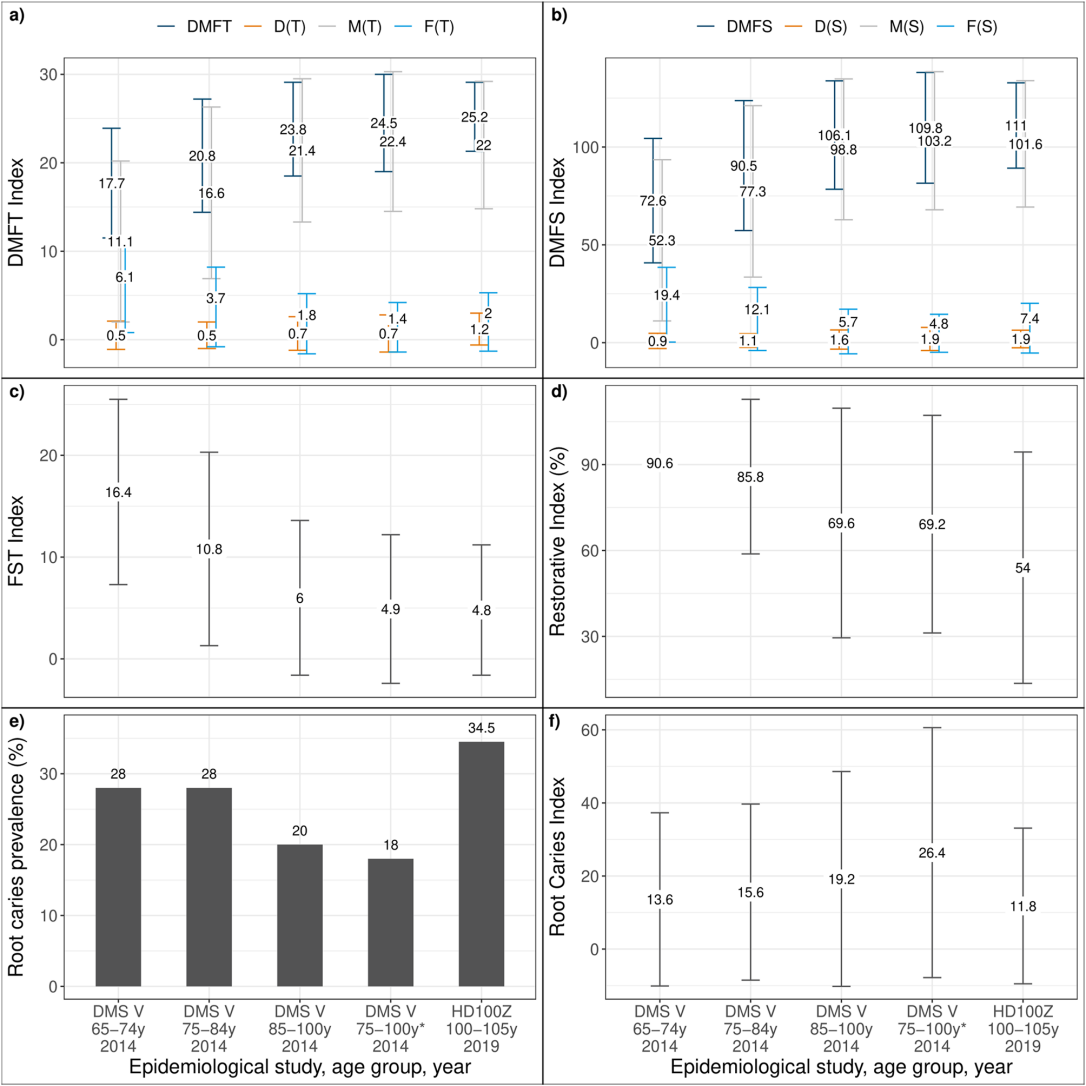
Mean oral health indices in older adults in Germany aged 75–100-years old and in the centenarian population of this study. (**a**) DMFT Index (**b**) DMFS-Index (**c**) FST-Index (**d**) Restorative Index (**e**) Root caries prevalence (**f**) Root Caries Index (standard deviation represented in bars if available).

Figure 2a shows a comparison of the DMF-T Index of centenarians and of adults aged 75–100 years. The 75–84-year-olds and 85–100-year-olds examined in the DMS V had a mean DMF-T of 20.8 and 23.8, respectively. Accordingly, centenarians presented with a DMF-T higher than in all younger age groups of older adults previously examined. However, differences are relatively small when centenarians are compared to those older adults with a recognized nursing care level (24.5 vs. 25.2 DMF-T). Here, centenarians even presented with less missing teeth but more decayed and filled teeth. These results are also confirmed by the DMF-S values. The mean DMF-S of centenarians was 111.0 ± 21.8, with a mean D(S) of 1.9 ± 4.5, mean M(S) of 101.6 ± 23.3 and F(S) of 7.4 ± 12.7. The DMF-S was therefore higher than in any younger age group previously examined in the DMS V (90.5 DMF-S in 75–100-year-olds, 106.1 DMF-S in 85–100-year-olds, 109.8 DMF-S in 75–100-year-olds in need of nursing care). Compared to the latter, centenarians presented with less missing surfaces but still more decayed and filled surfaces (Fig. 2b).

The mean number of teeth with root caries in dentate centenarians was 1.1 ± 1.5. Sex, educational level, residence, the presence of a recognized nursing care level or the utilization of dental services showed no influence. At least one tooth with root caries was found in 34.5% of centenarians (Fig. 2e), which is a relative increase in root caries prevalence of 32.7% compared to the root caries prevalence of 26.0% in 75–100-year-olds. However, the Root Caries Index (RCI) of centenarians was lower, amounting to 11.8% versus 16.4% in 75–100-year-olds. It was even lower than in older adults aged 65–74 years (see Fig. 2f for details).

### Restorative index

The Restorative Index indicates how restorative treatment needs are met; its maximum level is set at 100%. Centenarians presented with a mean Restorative Index of 54.0% compared to 83.0% in the group of 75–100-year-olds. This is 16 percentage points lower than in 85–100-year-olds and 32 percentage points lower than in 75–84-year-olds. Accordingly, an upwards trend in treatment deficiencies must be noted (Fig. 2d).

A similar trend is apparent when regarding the functioning of centenarians’ dentitions (Fig. 2c). The FST Index was considerably lower in centenarians than in those aged 75–100 years. However, it is almost equal to the FST of those older adults already in need of nursing care (4.9 in 75–100-year-olds with nursing care, 4.8 in centenarians). Men had more filled and sound teeth than women (9.0 vs. 4.0, *p* = 0.016). There were no statistically significant differences in the FST regarding the educational level, residence, the presence of a recognized nursing care level in centenarians or their utilization of dental services.

### Functional capacity and care needs

**Figure 3:**
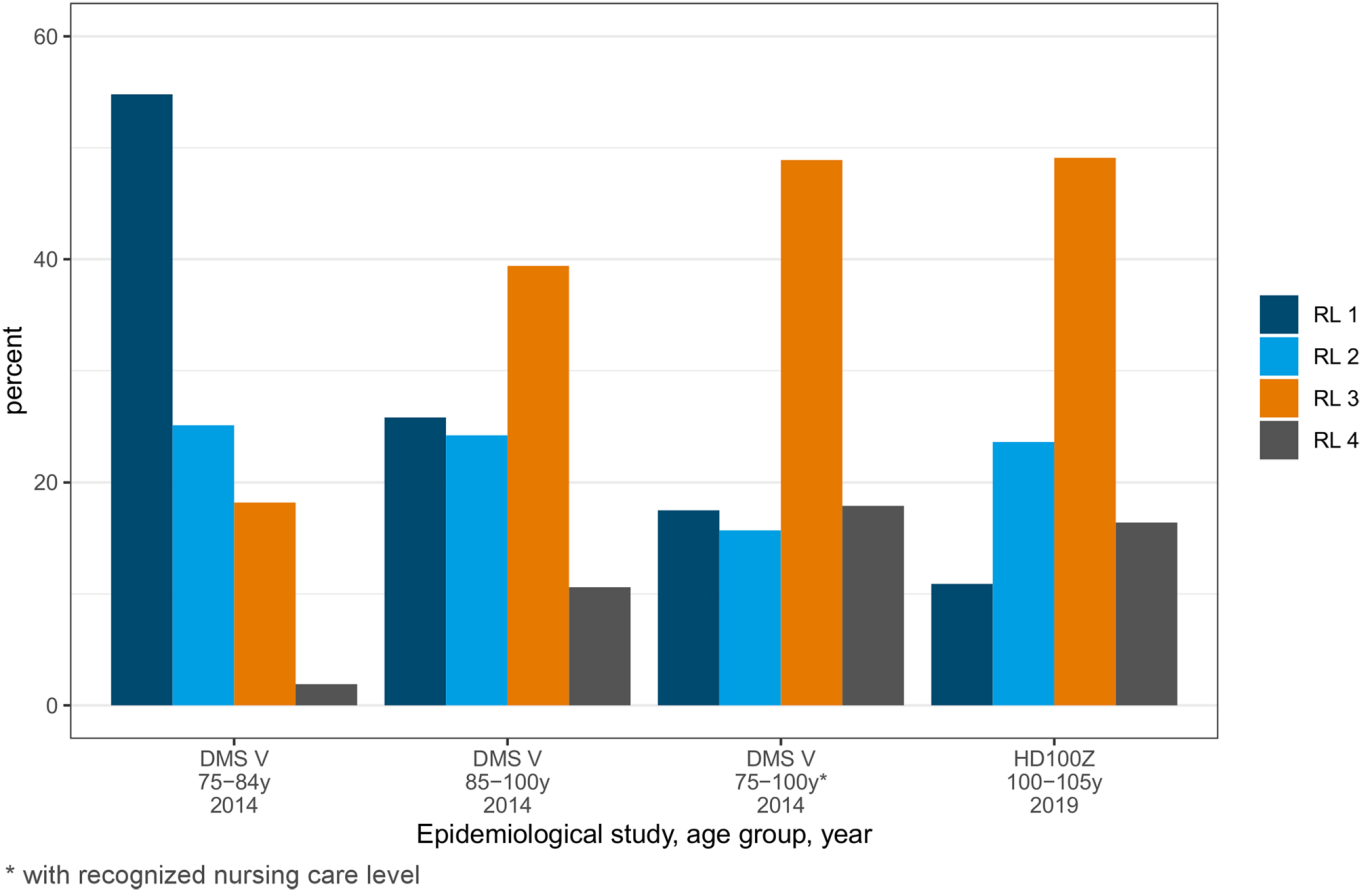
Resilience level in older adults in Germany aged 75–100 years old and in the centenarian population of this study.

Centenarians’ functional capacity was considerably lower than in those aged 75–100 years. 63.7% were not treatable or greatly reduced in their treatment capabilities, as defined by Nitschke et al.^34^. In contrast, only 19.4% of 75–100-year-olds were classified into these categories (see Table 2 for further details on the different age groups). Similar conditions can be noted when regarding the capability of oral hygiene, 43.6% showed greatly reduced or non-existent capabilities to perform their oral hygiene. A majority of centenarians could still express the wish to visit a dentist (94.5%) but only two thirds could organize the visit themselves. Grouped into resilience levels, 65.5% reached level 3 or 4 and were therefore hardly able to utilize dental services (Fig. 3). Centenarians with lower educational level (*p* = 0.018), living in a care facility (*p* = 0.045) or in need of nursing care (*p* = 0.001) were more likely to have a low functional capacity. Sex or the type of utilization of dental services had no significant influence.

**Table 2:** Functional capacity.

	Centenarian population	DMS V (85-100y)	DMS V (75-84y)
	n (%)	n (%)	n (%)
**Capability of treatment**			
Normal	7 (12.7)	98 (34.8)	535 (62.9)
Slightly reduced	13 (23.6)	79 (28.0)	201 (23.6)
Greatly reduced	26 (47.3)	91 (32.3)	106 (12.5)
Non-existent	9 (16.4)	14 (5.0)	9 (1.1)
**Capability of oral hygiene**			
Normal	10 (18.2)	94 (33.3)	517 (60.8)
Slightly reduced	21 (38.2)	107 (37.9)	260 (30.6)
Greatly reduced	19 (34.6)	66 (23.40)	65 (7.6)
Non-existent	5 (9.1)	15 (5.3)	9 (10.6)
**Autonomy**			
Normal	35 (63.6)	163 (57.8)	719 (84.5)
Reduced	17 (30.9)	90 (31.9)	117 (13.7)
Non-existent	3 (5.6)	29 (10.3)	15 (1.8)

Most centenarians received help in their daily activities (see online Appendix Supplementary Table S2 for details). 98.2% received help with household activities. Regarding personal hygiene, 69.1% received help showering or bathing, 50.9% received help washing and 27.3% received help using the toilet. 25.5% received help cutting their food. However, assistance in cleaning their teeth or dentures ranked last, and could only be observed in 12.7% of cases. Less than a third of centenarians deemed incapable to dress or undress themselves received help in brushing their teeth once or twice a day.

## Discussion

This is the first study world-wide that provides clinical information on the oral status and functional capacity of very old people with a mean age of 101.2 years. Our analysis shows that people born in 1919 and earlier still have remaining teeth. Although the findings from this target population, living in an area in South Germany, may not be transferrable to all populations, these results show how demographic changes might affect dental care in high-income countries.

Compared to younger groups, aged 75–100 years old, most clinical measures showed a decline in oral health of centenarians, with the exception of a remarkably low RCI. However, it must be clarified that a time span of 20 years lies between those two age groups. The decline of oral health is likely age-related and due to the lack of preventive oral health care during the childhood and early adult years of that generation^37,38^. Compared to previous centenarian studies based on self-reported information, the edentulous rate of 36.4% is very similar to the 36.5% rate reported in the US-American study by Kaufman et al.^16^ but is considerably lower than the 83% rate reported in the Netherlands^18^. Considering that all three countries are high income countries with a similar level of healthcare, the substantial differences may be due to variations in survey questions. For instance, in case of the questionnaire provided by Beker et al.^18^, a centenarian with an overdenture might incorrectly choose the option “removable complete denture” instead of reporting natural teeth in combination with a removable partial denture. This highlights the difficulties when using self-reported information.

With regard to health care and maintenance, required nursing care was highly prevalent in the age group 100+. Surprisingly, most clinical variables were not influenced by the question of institutionalization and nursing care. However, as an overwhelming majority of centenarians received nursing care, whether at home or in a care facility, this may have masked the previously often stated difference in the oral health status of institutionalized versus non-institutionalized older adults (Table 1)^39^. Our results showed similarities in the DMF-T, DMF-S, FST and resilience levels of centenarians and 75–100-year-olds in need of nursing care and therefore indicate this association (Figs. 2, 3). Most centenarians received help for daily activities, however, it must be stressed that oral health received little attention. Assistance in daily oral care was of least priority, even though many centenarians showed greatly reduced or non-existent capabilities to perform their oral hygiene. Receiving help going to the toilet and cutting food reached values twice as high, although motor functions necessary for sufficient oral hygiene are more intricate than those for the previously mentioned activities. There seems to be a lack of awareness amongst caregivers that the assisted person can no longer be solely responsible for his or her own oral hygiene.

The functional capacity of centenarians was reduced, and influenced by lower educational level, living in a care facility or the need of nursing care. It could be shown that only few centenarians still had all therapeutic options open to them, over 65% could no longer undergo regular dental treatments (reduced capability of treatment according to Nitschke et al.^34^) due to their reduced cognitive and physical condition. This is also highlighted by the exceptionally low Restorative Index. Moreover, a third of centenarians had not been seen by a dentist for over 5 years, including many participants residing in care facilities. Although some co-operations between care facilities and nearby dental practices exist, visits are still often scheduled only when a resident expresses the wish to consult a dentist, and these visits subsequently decline when the person is no longer able to do so. Due to the afore mentioned difficulties undergoing dental procedures, an increase in treatment by improving the reported deficiency of regular dental check-ups is questionable. However, visits by dental personnel may also improve oral health by motivation and education of the patient, and also of nursing staff in care facilities^40^. In contrast, GP-services seem to be adequate in quantity and quality^41^.

As a first of its kind, a limitation of this study is its exploratory character and comparisons with other scientific data are therefore limited. The population-based sample is only representative of a defined geographical region. Comparisons with the DMS V data must therefore be interpreted with caution. Moreover, though the gender distribution amongst centenarians is representative of this age group, the small sample size of 9 men limits gender differences observed. Furthermore, although the achieved response rate of 13% is substantial considering the difficulties contacting and motivating individuals aged 100 years or older to participate in a clinical study, the non-response bias is a further limitation of this study. A healthy volunteer effect may have biased the results towards a more favorable oral health status among the centenarian participants relative to the total population of centenarians. The fact that all centenarians were German citizens, thus subjected to nutritional deficits and deprivation following the turmoil of World War I and World War II, as well as the low capacities of dental care in those days, might have biased the results in a contrary effect.

## Conclusion

This study is first to give a detailed insight into the oral status of centenarians and supercentenarians in Germany. In summary, although a majority of centenarians still has remaining natural teeth, a decline of oral health compared to epidemiological data from older adults aged 75–100 years old is shown. This poses a problem, as we demonstrate that most centenarians no longer possess the functional capacity to undergo dental treatment at that age. Moreover, although most need care, assistance in daily oral health care is rare at present, and knowledge or compliance with recommended behaviors seems limited. Therefore, the results of this study may create an incentive to promote preventive measures and support in order to preserve oral health in the older population.

## Supplementary Information

Supplementary Information.
