# Supplementary material for: Oral health and functional capacity of centenarians
Source: Sci Rep. 2020 Dec 17;10:22215. doi: 10.1038/s41598-020-78842-w (PMC7747698; doi:10.1038/s41598-020-78842-w)

**Appendix**

**Oral health and functional capacity of centenarians**

Caroline Sekundo ^a*^, Eva Langowski ^a^, Samuel Kilian ^b^, Cornelia Frese ^a^

^a^ Clinic for Oral, Dental and Maxillofacial Diseases, University Hospital Heidelberg,

Department of Conservative Dentistry, Heidelberg, Germany

^b^ Institute of Medical Biometry and Informatics, University of Heidelberg, Heidelberg, Germany

* Corresponding author

Caroline Sekundo

Clinic for Oral, Dental and Maxillofacial Diseases, University Hospital Heidelberg

Department of Conservative Dentistry

Im Neuenheimer Feld 400

69120 Heidelberg, Germany

Tel: +49 6221 5639889

Fax: +49 6221 565074

E-mail: caroline.sekundo@med.uni-heidelberg.de

| **Table S1**. Dental health behaviors and beliefs | | | | |
| --- | --- | --- | --- | --- |
|  | Centenarian population | | DMS V (85-100y) | DMS V (75-84y) |
|  |  | n (%) | n (%) | n (%) |
| Personal responsibility for dental health* | Low | 13 (30.2) | 13 (4.6) | 37 (4.4) |
|  | Medium | 21 (34.9) | 73 (26.1) | 212 (25.0) |
|  | High | 15 (34.9) | 194 (69.3) | 599 (70.6) |
| Frequency of tooth brushing | At least 3 times daily | 11 (20.0) | 60 (21.3) | 142 (16.7) |
|  | Twice a day | 17 (30.9) | 138 (48.9) | 513 (60.4) |
|  | Once a day | 21 (38.2) | 71 (25.3) | 169 (19.9) |
|  | Several times a week | 0 | 5 (1.8) | 16 (1.9) |
|  | Once a week | 5 (9.1) | 0 | 1 (0.1) |
|  | Less than once a week | 0 | 4 (1.4) | 2 (0.2) |
|  | Never | 1 (1.8) | 4 (1.4) | 7 (0.8) |
| Utilization of dental services | Control-oriented | 27 (50.9) | 138 (49.3) | 556 (65.6) |
|  | Complaint-oriented | 26 (49.1) | 142 (50.7) | 292 (34.4) |
| Last dental visit | In the last year | 28 (50.9) | 177 (63.0) | 660 (77.5) |
|  | In the last two years | 6 (10.9) | 38 (13.6) | 81 (9.5) |
|  | In the last five years | 5 (9.1) | 22 (7.9) | 43 (5.0) |
|  | More than five years ago | 16 (29.1) | 44 (15.5) | 68 (8.0) |
| *How much can one do to preserve or improve one’s dental health? | | | | |

| **Table S2**. Help/ nursing care received for daily activities (n=55) | |
| --- | --- |
| Activity | n (%) |
| Household (cooking, cleaning, shopping) | 54 (98.2) |
| Leaving the residence for visits to the doctor/dentist | 51 (92.7) |
| Showering/bathing | 38 (69.1) |
| Washing | 28 (50.9) |
| Dressing/undressing | 26 (47.3) |
| Getting up/going to bed | 19 (34.6) |
| Toilet | 15 (27.3) |
| Cutting food | 14 (25.5) |
| Combing/shaving | 8 (14.6) |
| Oral hygiene | 7 (12.7) |

**Figure S1** Extraoral and intraoral view of a 102-year-old male patient. a. extraoral view during smiling, b. intraoral view during habitual occlusion, c. upper jaw, d. lower jaw


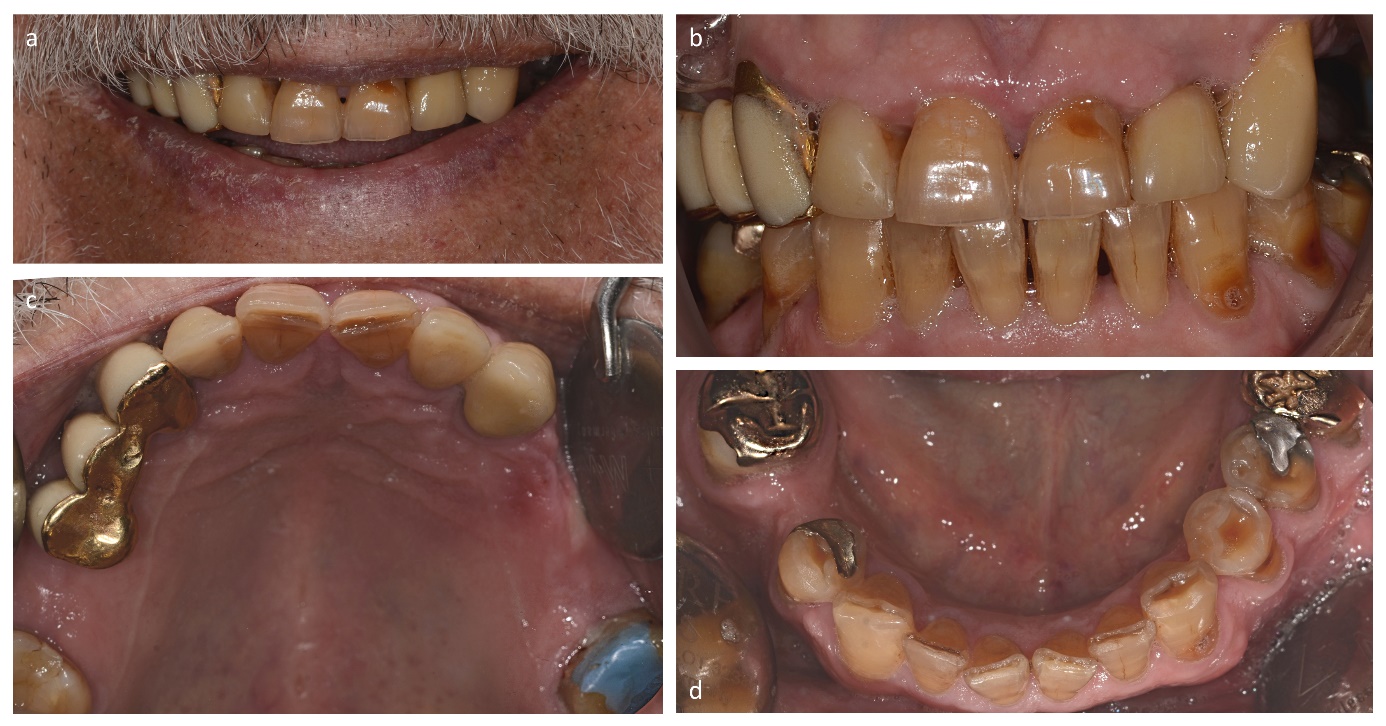

Supplement: Supplementary file 1 — Supplementary Information. [file 41598_2020_78842_MOESM1_ESM.docx]
